# *Klebsiella pneumoniae* ST147 harboring *bla*_NDM-1_, multidrug resistance and hypervirulence plasmids

**DOI:** 10.1128/spectrum.03017-23

**Published:** 2024-02-05

**Authors:** Frederick Ofosu-Appiah, Ezra E. Acquah, Jibril Mohammed, Comfort Sakyi Addo, Bright Agbodzi, Dorcas A. S. Ofosu, Charles J. Myers, Quaneeta Mohktar, Opoku-Ware Ampomah, Anthony Ablordey, Nana Ama Amissah

**Affiliations:** 1 West African Centre for Cell Biology of Infectious Pathogens, University of Ghana, Accra, Ghana; 2 Department of Bacteriology, Noguchi Memorial Institute for Medical Research, University of Ghana, Accra, Ghana; 3 The Burns Unit, Reconstructive Plastic Surgery and Burns Unit, Korle Bu Teaching Hospital, Accra, Ghana; Yangzhou University, Yangzhou, China

**Keywords:** *Klebsiella pneumoniae*, multidrug resistance, *bla*_NDM-1 _gene, hypervirulence, mobile genetic elements, burn wounds

## Abstract

The spread of hypervirulent (hv) and carbapenem-/multidrug-resistant *Klebsiella pneumoniae* is an emerging problem in healthcare settings. The New Delhi metallo-β-lactamase-1 (*bla*_NDM-1_) is found in Enterobacteriaceae including *K. pneumoniae.* The *bla*_NDM-1_ is capable of hydrolyzing β-lactam antibiotics which are used for treatment of severe infections caused by multidrug-resistant Gram-negative bacteria. This is associated with the unacceptably high mortality rate in immunocompromised burn injury patients. This study reports on the characterization of *bla*_NDM-1_ gene and virulence factors in hv carbapenem-/multidrug-resistant *K. pneumoniae* ST147 in the burns unit of a tertiary teaching hospital during routine surveillance. Two *K. pneumoniae* strains were obtained from wounds of burn-infected patients from May 2020 to July 2021. The hypervirulence genes and genetic context of the *bla*_NDM-1_ gene and mobile genetic elements potentially involved in the transposition of the gene were analyzed. We identified a conserved genetic background and an IS*26* and open reading frame flanking the *bla*_NDM-1_ gene that could suggest its involvement in the mobilization of the gene. The plasmid harbored additional antibiotic resistance predicted regions that were responsible for resistance to almost all the routinely used antibiotics. To ensure the identification of potential outbreak strains during routine surveillance, investigations on resistance genes and their environment in relation to evolution are necessary for molecular epidemiology.

**IMPORTANCE:**

Data obtained from this study will aid in the prompt identification of disease outbreaks including evolving resistance and virulence of the outbreak bacteria. This will help establish and implement antimicrobial stewardship programs and infection prevention protocols in fragile health systems in countries with limited resources. Integration of molecular surveillance and translation of whole-genome sequencing in routine diagnosis will provide valuable data for control of infection. This study reports for the first time a high-risk clone *K. pneumoniae* ST147 with hypervirulence and multidrug-resistance features in Ghana.

## INTRODUCTION

*Klebsiella pneumoniae* is an important human pathogen that causes nosocomial and community-acquired infectious diseases including septicemia and respiratory, urinary tract, skin and soft tissue and pneumonia infections (1). Over time, *K. pneumoniae* has acquired virulence factors and antibiotic resistance important for its pathogenesis (2–4). These genetic features of *K. pneumoniae* have been identified from multiple sources such as pulmonary infection and wounds, and have been associated with dysbiosis in the gut microbiome and changes in lung metabolome (5, 6). The global emergence of New Delhi metallo-β-lactamase-1 (*bla*_NDM-1_), a class B (zinc metallo-) β-lactamase, after its first detection in Sweden in 2008 from an Indian patient, is a major public health concern (7–9). First discovered in *K. pneumoniae* and *Escherichia coli*, New Delhi metallo-β-lactamase-1 (*bla*_NDM-1_) has been identified in other Enterobacteriaceae including *Citrobacter freundii*, *Enterobacter cloacae*, *Morganella morganii*, and *Proteus vulgaris* (10, 11). The *bla*_NDM-1_-positive Enterobacteriaceae has been found in both hospital and community-acquired infections, food, and water sources (12). This has led to the emergence of hypervirulent (hv) and carbapenem and/or multidrug-resistant *K. pneumoniae* spp. that cause invasive infections including endophthalmitis and meningitis in immunocompromised patients (13). Hypervirulent *K. pneumoniae* (hv-KP) is associated with a variety of factors including capsular serotypes, hypermucoviscosity, sequence types (ST11 and ST23), pathogenicity island, virulence plasmid, and virulence factors such as lipopolysaccharide, type VI secretion system, siderophore production and allantoin metabolism (14). Hv-KP pathogens are responsible for pyogenic liver abscesses, osteomyelitis and endophthalmitis in younger populations in the community (15). Over 78 capsular serotypes of *K. pneumoniae* have been reported; K1 and K2 serotypes are the most prevalent in hv-KP (16–19). Most hv-KP strains are susceptible to the commonly prescribed antibiotics, including carbapenems (20). However, these strains can acquire carbapenem or multidrug resistance that can lead to the emergence of isolates that have combined resistance and virulence. Alternatively, multidrug-resistant *K. pneumoniae* strain can acquire virulence plasmids (pLVPK/pVir-CR-HVKP4) (13, 21, 22). This has compromised the options for the treatment of life-threatening infections caused by *K. pneumoniae* with combined multidrug/carbapenem and hypervirulence factors (23).

First reported in Taiwan, hv-KP is observed in many Asian, European, and American countries, with Asian countries being endemic for several cases (24–27). *K. pneumoniae* with combined multidrug resistance and hypervirulence factors have emerged in a few African countries including Algeria, Egypt, Kenya, and Sudan (28–32). Carbapenem-/multidrug-resistant hv-KP have been reported in ST11, ST14, ST23, ST25, ST43, ST65, ST86, ST231, ST375, ST380, and ST1764, and are associated with *bla*_NDM-1_ and *bla*_KPC-2_ (33–38). In Ghana, Labi et al. (39) reported that *K. pneumoniae* made up 49.7% of the total microorganisms of the 1.0 incidence of bloodstream infections in hospitalized neonates per 100 person days. Furthermore, multidrug-resistant *K. pneumoniae* incidence contributed to 3.1% of rectal flora in patients undergoing prostate biopsy (40).

Bacteria that cause invasive wound infection can lead to systemic sepsis. It is important to report on the pathogenic potential of bacteria that cause invasive wound infection in burn patients. This study characterized the genetic environment of the plasmid-borne *bla*_NDM-1_ and hypervirulence genes in *K. pneumoniae* ST147 obtained from the burns unit of a tertiary care hospital in Ghana. Data analyses revealed that other mechanisms may initiate transposition of the *bla*_NDM-1_ than cointegration of IS*26* into the adjacent IS*26* region. The information obtained together with the phenotypic data will be valuable in routine surveillance and will guide infection prevention and control in our health institutions to prevent possible outbreaks.

## RESULTS

### Metadata from hv multidrug-resistant *K*. *pneumoniae* strains

Metadata of patients who carried isolates 016W_16082020 and 017WC1_20082020 are presented in Table 1. Patient 016 was prescribed cefuroxime, and patient 017 was prescribed amoxiclav and cefuroxime. Antibiotic susceptibility test revealed both strains displayed multidrug-resistant phenotypes (Table 2). All isolates were phenotypically resistant to commonly used antibiotics for treatment of *K. pneumoniae* infections in Ghana, including amikacin, ampicillin, ampicillin/sulbactam, aztreonam, cefepime, ceftazidime, cefotaxime, cefuroxime, ciprofloxacin, doripenem, imipenem, meropenem, piperacillin, and tobramycin, and susceptible to gentamicin, tigecycline, and trimethoprim/sulfamethoxazole.

**TABLE 1:** Metadata of patients colonized with hv carbapenem-/multidrug-resistant *K. pneumoniae*

Patient information	016	017
Age (years)	46	2
Gender	Female	Female
Total body surface area (%)	23	24
Cause of injury	Gas	Hot soup
Source of isolation	Wound	Wound
Antibiotics prescribed	Cefuroxime	Cefuroxime and amoxiclav
Duration of stay (days)	5	11
Time of admission		
Patient outcome	Death	Death

**TABLE 2:** Antimicrobial susceptibility test of hv carbapenem-/multidrug-resistant *K. pneumoniae* isolates^*a*^

Antibiotics	016W_16082020		017WC1_20082020	
	DD/mm	MIC/µg/mL	DD/mm	MIC (μg/mL)
Amikacin	15 < I	32	15 < I	32
Amp/sulbactam		>16/8		>16/8
Amoxiclav/30 µg	6 < R		6 < R	
Ampicillin		>16		>16
Aztreonam		>16		>16
Cefepime	10 < R	>16	10 < R	>16
Cefotaxime	6 < R	>16	6 < R	>16
Ceftazidime/30 µg	6 < R	>32	6 < R	>32
Cefuroxime/30 µg	6 < R	>16	6 < 6	>16
Cephazolin/30 mg	6 < R		6 < R	
Chloramphenicol/30 mg	6 < R		23 < S	
Ciprofloxacin/5 mg	6 < R		6 < R	
Clindamycin/ 2 µg	6 < R		6 < R	
Colistin/ 10 µg	13 < I		13 < I	
Doripenem/10 µg	16 < R	>2	17 < R	>2
Erythromycin/15 mg	6 < R		6 < R	
Fusidic acid/10 mg	6 < R		6 < R	
Gentamicin	18 > S	≤4	18 > S	≤4
Imipenem/10 µg	6 < R	>8	6 < R	>8
Kanamycin/30 mg	6<, R		6 < R	
Meropenem		>8		>8
Metronidazole/50 mg	6 < R		6 < R	
Nalidixic acid/30 mg	6 < R		6 < R	
Norfloxacin/10 mg	6 < R		6 < R	
Piperacillin		>64		>64
Rifampicin/5 mg	6 < R		6 < R	
Tetracycline/30 mg	6 < R		22 < S	
Tigecycline		≤2		≤2
Tobramycin/10 mg	6 < R	>8	6 < R	>8
Trimeth/sulfamethoxazole	17 > S	≤2/38	18 > S	≤2/38

^*a*^ Abbreviations: DD, disk diffusion; MIC, minimum inhibitory concentration; Trimeth, trimethoprim.

### *K. pneumoniae* ST147 phylogeny and genetic environment of *bla*_NDM-1_ and *bla*_oxa-1_ genes

**Fig 1:**
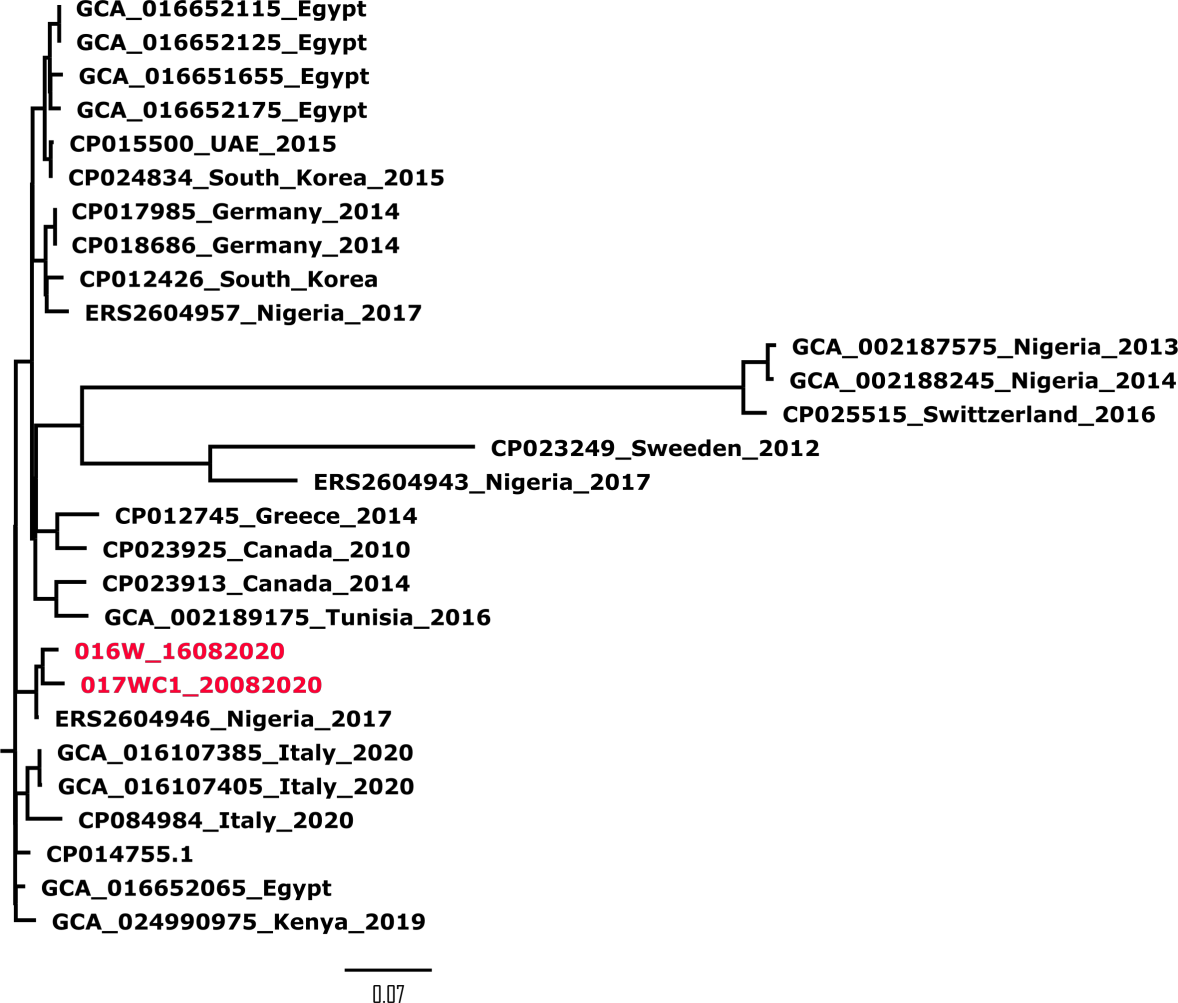
Phylogenetic analyses of *K. pneumoniae* ST147 strains. The Ghana strains 016W_16082020 and 017WC1_20082020 are colored in red. Most strains are labeled with their GenBank accession, country of origin, and year of isolation. The phylogenetic tree was constructed using CSIPhylogeny pipeline and visualized in FigTree.

**Fig 2:**
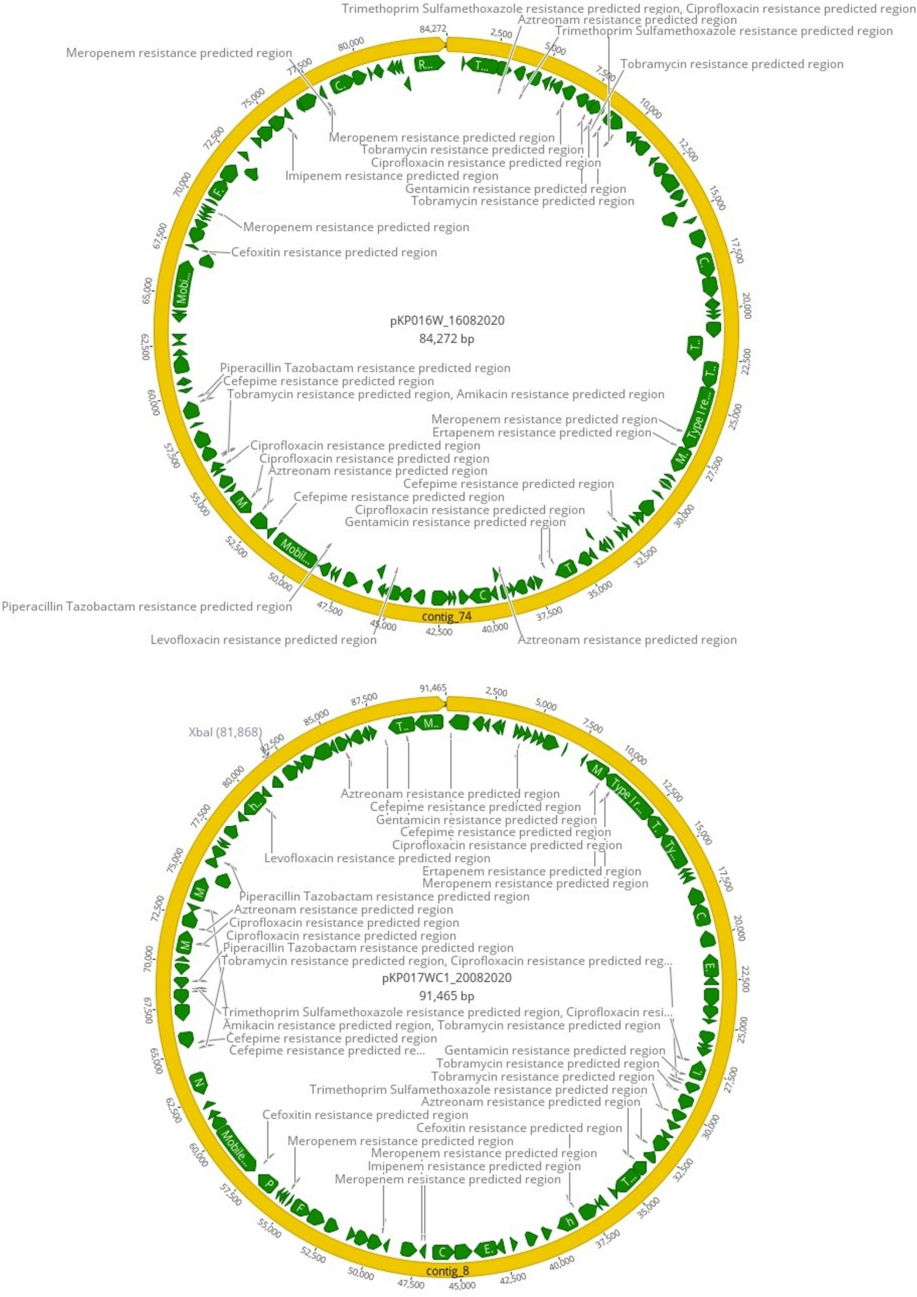
Schematic representation of plasmid bearing the *bla*_NDM-1_ gene and other antibiotic-resistant predicted regions in *K. pneumoniae* strains (top) 016W_16082020 and (bottom) 017WC1_20082020.

**Fig 3:**
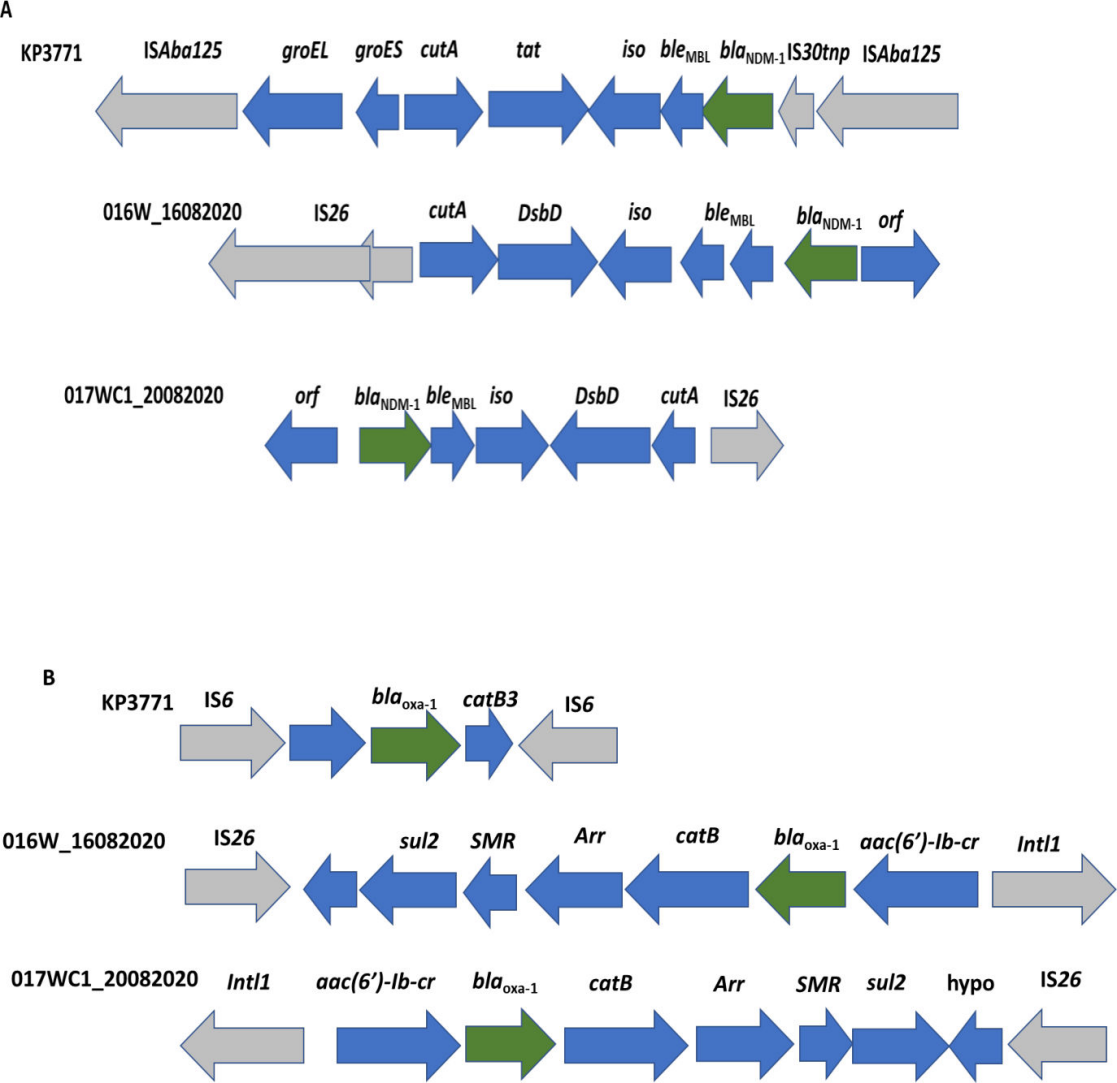
Genetic environment of beta-lactamase genes in *K. pneumoniae* strains. (**A**) Genetic structure of *bla*_NDM-1_ gene in the genomes of 016W_16082020 and 017WC1_20082020 strains in comparison with KP3771 reference strain. (**B**) Genetic structure of the *bla*_oxa-1_ gene in the genomes of 016W_16082020 and 017WC1_20082020 strains in comparison with KP3771 reference strain. The colored arrows denote the following: gray, mobile genetic elements including ISs and class 1 integron; green, *bla*_NDM-1_ gene; blue, antibiotic resistance genes and hypothetical genes.Abbreviations: *arr*, rifampin ADP ribosyl transferase; hypo, hypothetical gene; SMR, small multidrug-resistance efflux transpoter.

Isolates 016W_16082020 and 017WC1_20082020 were assigned to ST147. A phylogenetic tree was constructed using the whole-genome single nucleotide polymorphism (SNP) phylogeny approach to compare the genetic relatedness of our isolates with other global ST147 *K. pneumoniae* strains (Fig. 1). Isolates 016W_16082020 and 017WC1_20082020 clustered with a Nigerian strain ERS2604946. The isolates differed by 58 and 72 SNPs, respectively. Both isolates harbored similar plasmids: IncQ and IncF and an additional plasmid IncH1 for isolate 017WC1_20082020. We compared their genetic environment to assess whether the *bla*_NDM-1_ gene from both isolates originated from a similar source and were genetically related. The *bla*_NDM-1_ gene was located on untyped plasmid pKP016W_16082020 and pKP017WC1_20082020 with plasmid sizes of 84,272 and 91,465 bp, respectively (Fig. 2). The two isolates shared a highly conserved backbone structure (Fig. 3A); however, the *bla*_NDM-1_ gene of isolate 017WC1_20082020 was oriented in the opposite direction. Both isolates were flanked upstream by an open reading frame (ORF) and downstream by the putative ancestral genes: *ble*_MBL_-*iso-DsbD-cutA* (Fig. 3A). These genes code for bleomycin resistance, phosphoribosylanthranilate isomerase, protein disulfide reductase, and heavy metal resistance. This segment was followed by an insertion sequence IS*26*. However, isolate 016W_16082020 had two copies of IS*26* and *ble*_MBL_ compared to 017WC1_20082020. The two isolates lacked the IS*Aba125*, *groEL*, *groES*, *tat*, and IS*30tnp* genes when compared with KP3771 reference strain (Fig. 3A). The genetic environment surrounding the *bla*_oxa-1_ gene of both isolates shared a similar structure but in the opposite orientation. The *bla*_oxa-1_ gene was located with other resistance genes, *bla*_oxa-1_-*catB-Arr-SMR-sul*2-IS*26* downstream for isolate 016W_16082020. Upstream contained class 1 integron with the structure of *intl1-aac*(6′)*-Ib-cr-bla*_oxa-1_ (Fig. 3B), while the *bla*_oxa-1_ gene of the KP3771 reference strain was flanked by the composite IS*6* insertion sequence. For the class A beta-lactamase gene *bla*_CTX-M15_, it was bound upstream by the IS*2* and downstream by aminoglycoside *aac*(6′)*-Ib/aac*(6′)*-II* and *bla*_TEM_ (data not shown).

The pKP016W_16082020 (Fig. 2, top) and pKP017WC1_20082020 (Fig. 2, bottom) plasmids harbored predicted antibiotic resistance regions that have been shown to confer resistance to amikacin, aztreonam, cefepime, ciprofloxacin, ertapenem, fosfomycin, gentamicin, imipenem, levofloxacin, meropenem, piperacillin-tazobactam, tetracycline, tobramycin, and trimethoprim-sulfamethoxazole. Both plasmids did not contain the conjugal transfer gene.

### Detection of antibiotic and virulence genes

The antibiotic resistance genes identified for both isolates coded for aminoglycosides, beta-lactams, carbapenems, cephalosporins, fluoroquinolones, phenicol, and sulfonamides (Table 3). The antibiotic resistance genes were located on untyped plasmids pKP016W_16082020 and pKP017WC1_20082020 and lacked the conjugal transfer gene (Fig. 2). A variety of hypervirulence-associated factors were identified in both *K. pneumoniae* strains. This includes capsule-associated genes (K64), lipopolysaccharides, type VI secretion system, fimbrial adhesin, virulence-associated regulators, siderophores (aerobactin, enterobactin, and yersiniabactin), allantoin, and efflux pumps (Table 4). Isolate 017WC1_20082020 had additional genes coding for lipopolysaccharide core biosynthesis and transport system: *rfbD* (o-antigen) and *wzzE*. These isolates tested negative for hypermucoviscosity according to the string test. The hypervirulence genes of both isolates were located on the IncQ plasmid, which harbored the conjugal transfer gene *tra*G.

**TABLE 3:** Antibiotic-resistant genes identified in hv carbapenem-/multidrug-resistant *K. pneumoniae* isolates

Strain	Penicillins	Aminoglycosides	Carbapenems	Cephalosporins	Porin	Fluoroquinolones	Phenicol	Sulfonamides
016W_16082020	*bla*_OXA-1_, *bla*_OXA-9_, *bla*_TEM-190_, *bla*_TEM-1D_, and *bla*_SHV-11_	*aac*(6′), a*ac*(6′)*-Ib-cr.aadA*	*bla*_NDM-1_	*bla*_CTX-M-15_	*ompK35*	*qnrS*1, *gyrA*-83I, and *parC*-80I	*catB3*	*Sul2*
017WC1_20082020	*bla*_OXA-1_, *bla*_OXA-9_, and *bla*_SHV-11_	*aac*(6′), *aac*(6’)-*Ib-cr.aadA*	*bla*_NDM-1_	*bla*_CTX-M-15_	*ompK35*	*qnrS*1, *gyrA*-83I, and *parC*-80I	*catB3*	*Sul2*

**TABLE 4:** Hypervirulence genes identified in hv carbapenem-/multidrug-resistant *K. pneumoniae* isolates

Isolates	Capsular poly-saccharides	Lipopolysaccharides	Type VI secretion system	Fimbria and adhesin	Virulence-associated regulators	Siderophores	Allantoin metabolism	Efflux pumps	Plasmids
016W_16082020	K64	*eptA/pmrC,* heptosyltransferase I, *lapB*, *lptA*, *lptB*, *lptC*, *lptD*, *lptE*, *lptF*, *lptG*, lipid A palmitoleoytransferase *pagP*, lipid A biosynthesis palmitoleoytransferase, lipid A 1-diphosphate, lipid A-disaccharide synthase, *msbA*, *ompR*, *rfaZ*, *rfbB*, lipid A lauroyl acyltransferase, lipid A-dissacharide synthase, O2a	T6SS component *hcp*, T6SS PAAR-repeat protein *tssA*, *tssB*, *tssC*, *tssE*, *tssF*, *tssG*, *tssH*, *tssJ*, *tssK*, *tssL*, *tssM*	*fimA*, *fimB*, *fimC*, *fimD*, *fimE*, *fimG*, *fimH*, *fimI*, type-1 fimbrial protein, fimbrial subunit, type IV fimbrial (*pilB* and *pilC*)	*msgA*, *ompS*, *phoU*, *prmD* (PhoPQ-*pmrD-pmrAB*)	*entS* (enterobactin exporter), enterobactin esterase, *feoB*, *feoA*, (*fyuA*, *psn*, pesticin receptor), *fepB*, *fepC*, *fepD*, *fepG*, *fieF*, *feoC*, *fur*, iron acquisition 2,3-dihydroxybenzoate, iron siderophore ABC transporter, *irp1*, *irp2*, *irp3*, *irp5*, *iutA*, yersiniabactin (*ybtA, ybtP*, *ybtQ*, and *ybtT*)	Allantoin, cytosine/purine/thiamine uracil	*ybhF*, *ybhG*, *ybhR*, *ybhS*	IncQ
017WC1_20082020	K64	*eptA/pmrC*, heptosyltransferase I, *lapB*, *lptB*, *lptC*, *lptD*, *lptE*, *lptF*, *lptG*, lipid A palmitoleoytransferase *pagP*, lipid A biosynthesis palmitoleoytransferase, lipid A 1-diphosphate, lipid A-disaccharide synthase, *msbA*, *ompR*, *rfaZ*, *rfbB*, r*fbD* (o-antigen), lipid A lauroyl acyltransferase, lipid A-dissacharide synthase, O2a, *wzzE*	T6SS component *hcp*, T6SS PAAR-repeat protein *tssA*, *tssB*, *tssC*, *tssE*, *tssF*, *tssG, tssH*, *tssJ*, *tssK*, *tssL*, *tssM*	*fimA*, *fimB*, *fimC*, *fimD*, *fimE*, *fimG*, *fimH*, *fimI*, type 1 fimbrial protein, fimbrial subunit, type IV fimbrial (*pilB and pilC*)	*msgA*, *ompS*, *phoU*, *prmD* (PhoPQ-*pmrD-pmrAB*)	*entS* (enterobactin exporter), enterobactin esterase, *feoB*, *feoA*, (*fyuA*, *psn*, pesticin receptor), *fepB*, *fepC*, *fepD*, *fepG*, *fieF*, *feoC*, *fur*, iron acquisition 2,3-dihydroxybenzoate, iron siderophore ABC transporter, *irp1*, *irp2*, *irp3*, *irp5*, *iutA*, yersiniabactin (*ybtA, ybtP*, *ybtQ*, and *ybtT*)	Allantoin, cytosine/purine/thiamine uracil	*ybhF, ybhG, ybhR, ybhS*	IncQ

## DISCUSSION

Routine surveillance of antimicrobial-resistant (AMR) pathogens is being widely explored in low- and middle-income countries (LMICs) to inform treatment outcomes. However, genomic surveillance to monitor bacterial evolution, antimicrobial resistance, and virulence determinants over time is relatively few in LMICs (21, 41–43). According to the World Health Organization’s list of AMR bacteria, *K. pneumoniae* has been reported as a high-priority antibiotic-resistant pathogen responsible for global nosocomial infections (44). Here, we report on the genetic environment of carbapenem-resistant *bla*_NDM-1_ gene and hypervirulence of *K. pneumoniae* obtained during routine surveillance in the burns unit of a tertiary teaching hospital in Ghana to gain insights into emerging high-risk clone ST147.

Extended-spectrum β-lactamase-producing *K. pneumoniae* ST13, ST15, ST22, ST25, ST36, ST70, ST110, ST147, ST334, ST405, ST414, ST502, and ST530 have been reported in bloodstream infections, poultry, and wounds of patients (45–47). Of these, ST147 has emerged as a high-risk clone reported in hospital outbreaks globally (48). Hospital outbreaks of *K. pneumoniae* ST147 have been reported in China (49), Greece (50), Slovenia (51), and Tunisia (52, 53). In Africa, cases have been reported from wound infections and community samples from Algeria (32, 54), Burkina Faso (55), Egypt (56), Kenya (57, 58), Libya (59), Nigeria (60), and Tunisia (61–64). Other sources such as animals from Senegal (65) and poultry products from Ghana (46) have been shown to harbor *K. pneumoniae* ST147. In a decade, there has been a global dissemination of ST147 high-risk clone. The close relatedness of our strains with a strain from Nigeria may suggest recent travel history of patients or contact with colonized persons in Ghana. The detection of ST147 underscores the importance of molecular surveillance in healthcare settings to prevent nosocomial outbreaks.

Carbapenem-/multidrug-resistant *K. pneumoniae* isolates were resistant to the prescribed antibiotics. The strains were also resistant to other antibiotics prescribed in the burns unit: ceftazidime, ciprofloxacin, imipenem, and meropenem except for gentamicin, tigecycline, and trimethoprim/sulfamethoxazole. Combination therapy for the treatment of carbapenem-resistant pathogens, including ceftazidime-avibactam, and azetreonam-avibactam, or monotherapy using cefiderocol is not the current protocol for the treatment of patients in the burns unit; hence, antimicrobial susceptibility test for these antibiotics was not tested. Due to the intermediate resistance observed, the last-resort antimicrobial, colistin, was not administered to the patients. Gentamicin and trimethoprim/sulfamethoxazole predicted resistance regions were detected; however, the strains were phenotypically susceptible at concentrations of ≤4 µg/mL and ≤2/38 µg/mL, respectively. The reasons for this observation were not explored in this study. Multidrug resistance leads to serious illness, prolonged hospital stays, and poor patient outcomes due to treatment failure. There is an urgent need to promote the appropriate use of antimicrobials to reduce microbial resistance. Hv-KP strains are highly pathogenic and known to cause invasive infections, specifically pyogenic liver abscess, meningitis, and endophthalmitis, in immunocompromised and healthy individuals (15, 66). Capsular serotypes including K5, K16, K20, K28, K54, K57, K63, K64 (identified in this study), and KN1 have been detected in hv-KP and associated with high pathogenicity and transmissibility (67, 68). The hv-KP acts by evading the host immune system, leading to severe invasive infections (19). Given the repertoire of virulence factors identified in the two carbapenem-resistant *K. pneumoniae* isolates, we envisage that the virulence factors could cause wound abscess, severe infection leading to sepsis, and multiple organ dysfunction syndrome, the primary cause of death in burn patients (69).

*K. pneumoniae* ST147 strains have acquired resistance genes on plasmids, including oxacillinases: *bla*_oxa-48_ on IncL (70), *bla*_oxa-181_ on the chromosome (71, 72), *bla*_oxa-204_ on IncA/C (73), metallo-beta-lactamase *bla*_NDM-1_ on IncFIIA (74), IncA/C (75), and IncX3 (76), and *bla*_KPCs_ on pKpQIL (77) and IncN (78). Several mobile genetic elements (MGEs) such as plasmids, integrons, and transposons are associated with acquiring and transmitting carbapenem resistance genes and other AMR determinants between bacteria (79, 80). Insertion sequences (ISs) and transposons including IS*Aba125*, IS*3000*, IS*26*, IS*5*, IS*CR1*, IS*CR27*, Tn*3*, Tn*125*, and Tn*3000* have played vital roles in the dissemination of *bla*_NDM_ genes. There has been a temporal role of plasmids, ISs, and transposons in the mobilization of *bla*_NDM_ genes. First Tn*125* and then Tn*3000* and IS*26* were involved in the transposition of the carbapenem gene (81, 82). In this study, the ST147 strains harbored the *bla*_NDM-1_ genes on plasmids pKP016W_16082020 and pKP017WC1_20082020. The IS*26* was found in two positions on both plasmids about 36–52 kb apart; the second IS*26* flanked *bla*_oxa-1_ in the opposite orientation, which suggests involvement in two independent activities of mobilization of antibiotic-resistant genes. Given that transposition of IS*26* with the *bla*_NDM-1_ gene occurs via cointegration into the adjacent IS*26* with the same orientation (83), it is unlikely that the transposition will occur successfully in our strains for the following reasons: (i) the *bla*_NDM-1_ gene was not flanked immediately on both sides by IS*26* to facilitate transposition of the *bla*_NDM-1_ gene, and (ii) the orientation of the second IS*26* is in the opposite direction compared to experiments shown to demonstrate mobilization of the *bla*_NDM-1_ gene. Since the *bla*_NDM-1_ gene is carried on non-conjugative plasmids, additional processes including recombination events may be involved in the transposition of the *bla*_NDM-1_ gene. Further studies are needed to improve our understanding of how IS*26* pseudo-composite transposon observed in isolate 016W_16082020 will mobilize the *bla*_NDM-1_ gene to adjacent IS*26* that is oriented in the opposite direction. Considering that the mobility of the *bla*_NDM-1_ gene is restricted due to the absence of the conjugal transfer gene and orientation of the second IS*26*, we suggest a combined role of the flanking IS*26* and the ORF gene in the dissemination of the *bla*_NDM-1_ gene. Alternatively, other means of transfer, including mobilization by the virulence conjugative plasmid, IncQ (helper plasmid), or transduction by a bacteriophage, could be used to transfer the carbapenem resistance genes to other bacteria. With the high number of predicted regions for AMR, further studies are needed to demonstrate the dissemination of these genes across different bacterial genera.

Our study characterized the genetic environment and virulence factors of hypervirulent carbapenem-/multidrug-resistant *K. pneumoniae* strains harboring *bla*_NDM-1_ on plasmids pKP016W_16082020 and pKP017WC1_20082020 and the MGEs involved in its complex evolutionary dissemination. This highlights the virulence potential and limited options for antimicrobial therapy of patients whose wounds are infected with hv carbapenem-/multidrug-resistant *K. pneumoniae* strains. It is important to implement genomic surveillance in low-resource settings to promptly identify and control the spread of carbapenem resistance and monitor evolving resistant bacteria in our healthcare settings.

## MATERIALS AND METHODS

### Cultivation of hv multidrug-resistant *K. pneumoniae* and antimicrobial susceptibility

Two *K. pneumoniae* strains, 016W_16082020 and 017WC1_20082020, were obtained from patients in the burns unit between May 2020 and July 2021. Clinical isolates were selected from these patients because their burn injuries were infected with hv carbapenem-/multidrug-resistant *K. pneumoniae*. The clinical samples were cultured on MacConkey agar (Oxoid Ltd, Basingstoke, UK) and incubated at 37°C for 24 hours with the reference strain *K. pneumoniae* ATCC 700603. Subcultures were made on nutrient agar (Oxoid Ltd) plates supplemented with 100-μg/mL imipenem-meropenem antibiotics. Identification was done using matrix-assisted laser desorption ionization-time of flight mass spectrometry with a microflex LT Biotyper v.3.0 (Bruker; Daltonics, Bremen, Germany) according to the manufacturer’s instructions.

The minimum inhibitory concentrations of amikacin, ampicillin, ampicillin/sulbactam, amoxicillin-clavulanate, aztreonam, cefepime, ceftazidime, cefotaxime, ceftriaxone, cefuroxime, ciprofloxacin, colistin, doripenem, ertapenem, gentamicin, imipenem, meropenem, nitrofurantoin, piperacillin-tazobactam, tigecycline, tobramycin, and trimethoprim/sulfamethoxazole were determined using the broth microdilution method (84) with the MicroScan autoSCAN-4 System (Beckman Coulter, Brea, CA, USA) according to the manufacturer’s instructions. The Kirby-Bauer disk diffusion method (85) was used to test the antimicrobial susceptibility of the isolates to confirm the previous test. The results were interpreted according to the Clinical and Laboratory Standards Institute 2020 guidelines (86). The string test was performed on both isolates for detection of hypermucoviscosity (87).

### Genomic sequencing and analysis

Genomic DNA of isolates was extracted using QIAamp DNA Mini Kit (Qiagen, Hilden, Germany) according to the manufacturer’s instructions and quantified using Qubit1 X dsDNA HS Assay Kit (ThermoFisher Scientific, MA USA) on the Qubit 4 Fluorometer (ThermoFisher Scientific). DNA libraries were prepared using Rapid barcoding (RQK-RBK004) and sequenced on the GridION MK1 sequencer (GXB03459) (Oxford Nanopore Technologies Ltd, Oxford Science Park, UK) (88–90). All procedures of DNA preparation, library construction, and genome sequencing were done according to the manufacturer’s instructions. The pass reads were trimmed off adapter sequences using Porechop v.0.2.4. Nanofilt v.1.0.5 was used to quality-filter trimmed reads to remove reads with average quality of <9 and length shorter than 500 bp. The resultant high-quality reads were used for *de novo* assembly using flye v.2.8.1 and polished with medaka v.1.6.1. The phylogenetic tree was constructed using the CSIPhylogeny pipeline (https://cge.food.dtu.dk/services/CSIPhylogeny/) (91). Bactinspector v.0.1.3 was used to select an appropriate reference (CP014755.1). The tree was visualized in FigTree, and SNP distances between strains were calculated using snp-dists v.0.8.2 (https://github.com/tseemann/snp-dists). The assembled data were annotated using RAST server platform (https://rast.nmpdr.org) (92) and analyzed using Geneious Prime v.2023.0.4. The global platform for genomic surveillance, Pathogenwatch (https://pathogen.watch/), was used to predict antibiotic resistance and virulence genes including the capsule serotype (K and O).

## Data Availability

The sequence reads were submitted to the National Center for Biotechnology Information GenBank and are available under the BioProject number PRJNA944783 and accession numbers JARNMI000000000 and JARNMH000000000.
